# Hematopoietic Effects of Paeoniflorin and Albiflorin on Radiotherapy-Induced Myelosuppression Mice

**DOI:** 10.1155/2016/5789381

**Published:** 2016-05-26

**Authors:** Yingli Zhu, Linyuan Wang, Zhihui Yang, Jingxia Wang, Wei Li, Jianyu Zhou, Jianjun Zhang

**Affiliations:** ^1^Beijing University of Chinese Medicine, Beijing 100029, China; ^2^Department of Psychiatry & Neuroscience, University of Florida, Gainesville, FL 32611, USA

## Abstract

*Paeonia lactiflora* root (baishao in Chinese) is a commonly used herb in traditional Chinese medicine (TCM). Paeoniflorin (PF) and albiflorin (AF) are two major active constituents of* P. lactiflora*. In this paper, we aimed to investigate the hematopoietic effects of PF and AF on myelosuppression mice induced by radiotherapy and to explore the underlying mechanism. The finding indicated that PF and AF significantly increased the numbers of white blood cells (WBC) and reversed the atrophy of thymus. Furthermore, PF and AF increased the levels of granulocyte-macrophage colony-stimulating factor (GM-CSF) and interleukin-3 (IL-3) and reduced the levels of tumor necrosis factor-*α* (TNF-*α*) in serum and increased the level of colony-stimulating factor (G-CSF) in plasma. Lastly, PF and AF not only enhanced the mRNA levels of GM-CSF and G-CSF in the spleens, but also increased the protein levels of G-CSF and GM-CSF in bone marrow. Our results suggest that PF and AF may promote the recovery of bone marrow hemopoietic function in a myelosuppressed mouse model.

## 1. Introduction

The root of* Paeonia lactiflora* Pall (family Ranunculaceae) is commonly used in China, Korea, and Japan.* P. lactiflora* is a component herb of many traditional formulae, such as Siwu-Tang, which has been widely used in treating palpitation, dysmenorrhea, chronic inflammation, and aminia [1]. There are a lot of major active constituents of* P. lactiflora* that have been identified, including paeoniflorin (PF), albiflorin (AF), oxypaeoniflorin, paeonilactinone, benzoyloxypaeoniflorin, and lactinolide [2]. It is well documented that PF possesses remarkable effects for pain [3, 4], muscle spasm [5, 6], inflammation [7, 8], and neurodegenerative disorders [9, 10]. However, few pharmacological studies of AF were reported. Most recently, we have found that PF and AF could suppress radiation and chemotherapy-induced myelosuppression [11–13]. In a recent report, an active fraction from* P. lactiflora* containing paeoniflorin and albiflorin (CPA) showed ameliorative effects on myelosuppression induced by radio and chemotherapy [14]. In another study, Jiang has proved the anti-inflammation effects of TGP on neutrophil cAMP-PDE activity in a rat arthritis model [6].

Blood/bone marrow system is one of the largest organs in the body that is an important potential target in ionizing radiation [15]. Acute exposure to elevated dose of ionizing radiation causes defects in hemopoiesis, resulting in low numbers of circulating blood cells, and increases susceptibility to infection [16]. Consequently, it has become a routine procedure in the investigation of hematological and bone marrow disorders in radiotherapy assessments. Nowadays, efforts to stimulate hematopoiesis in myelosuppression animals have involved hematopoietic cytokines [17], such as colony-stimulating factor (G-CSF), granulocyte-macrophage colony-stimulating factor (GM-CSF), interleukin-3 (IL-3), interleukin-6 (IL-6), and tumor necrosis factor-*α* (TNF-*α*), that can be used to accelerate hemopoietic recovery.

Consistent with our previous study [11–13], still, we hypothesized that PF and AF, two characteristic isomers in* P. lactiflora*, have the hematopoietic effect as well as the antimyelosuppression effect. Mechanisms associated with hematopoietic cytokines may also be involved in the hematopoietic effects of the two isomers. To test this hypothesis, we examined the effects of PF and AF in 3.5 Gy Co^60^ *γ*-rays irradiated-induced myelosuppression mice [18]. We also checked the hematopoietic functions of PF and AF, which are characterized by the changes of the number of blood cells, including WBC, RBC, and HGB. Moreover, considering the close relationship between immunity and hematopoiesis [15], the thymus index and spleen index were also investigated. To further determine the mechanism underlying the hematopoiesis activity of PF and AF, we analyzed the hematopoiesis-related cytokines in serum or in plasma as well as their mRNA in spleen and protein expressions in bone marrow cells. In this study, the effects of PF and AF on hematopoiesis in myelosuppression mice induced by radiotherapy were investigated systematically and the underlying mechanisms were also explored.

## 2. Materials and Methods

### 2.1. Chemical Compounds

**Figure 1:**
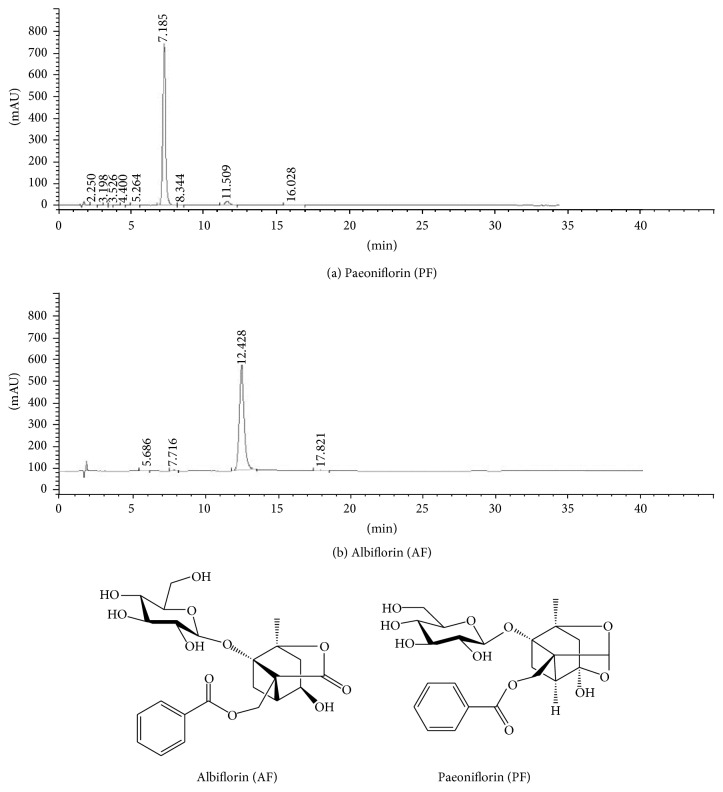
Chemical structures and HPLC chromatograms of paeoniflorin (PF) (a) and albiflorin (AF) (b).

Paeoniflorin (PF) and albiflorin (AF) were prepared in our library (patent number ZL 201110184287.4, China). The purity of PF (purity = 98.6%) and AF (purity = 96.7%) was measured by reverse-phase high-performance liquid chromatography (HPLC) coupled with ultraviolet detection according to Chinese Pharmacopoeia (2010, Beijing, China). The HPLC chromatogram and chemical structures of the two isomers are shown in Figure 1. PF and AF were separately dissolved in 0.9% normal saline and diluted to the desired concentration on the day of testing.

### 2.2. Animals

Kunming mice (male, 6 weeks old, 18–22 g) were obtained from the Vital River Co., Ltd. (Beijing, China) and maintained at controlled temperature (22 ± 2°C) and humidity (50 ± 10%) with a 12 h light/dark cycle. All efforts were made to minimize the pain of animals. The experimental protocol was approved by the Ethics Committee of Beijing University of Chinese Medicine (number Kj-dw-18-20150731-01).

### 2.3. The *γ*-Ray Treatment

This *γ*-ray treated myelosuppression mice model was set up according to Liang's method [16, 18]. After 7 days of acclimatization, mice were divided into six groups (*n* = 10 mice per group, males), including (1) normal control group (control), (2) *γ*-ray treated group (model), (3) high-dose PF treatment group (PF-H, 30 mg/kg PF), (4) L-dose PF treatment group (PF-L, 15 mg/kg PF), (5) high-dose AF treatment group (AF-H, 30 mg/kg AF), and (6) L-dose AF treatment group (AF-L, 15 mg/kg PF).

All mice except the control and model were intragastrically (i.g.) pretreated with the different doses of chemical compounds, PF (30 mg/kg and 15 mg/kg), and AF (30 mg/kg and 15 mg/kg) for 7 days. On the 8th day, mice except the normal group received total body irradiation of 3.5 Gy Co^60^ *γ*-rays at a dose rate of 1.60 Gy. After irradiation, control group mice were treated in exactly the same way as the irradiated animals; PF and AF were administered daily intragastrically at a dose of 0.2 mL/20 g body weight for 14 consecutive days. Normal and model mice were given normal saline.

### 2.4. Peripheral Leukocyte Count, Body Weight, Thymus Index, and Spleen Index

After the last administration, body weights of mice were measured. Then the mice were subjected to light diethyl ether anesthesia and blood was collected into clean test tubes with or without ethylenediaminetetraacetic acid (EDTA) by extracting eyeballs. A portion (200 *μ*L) of blood with EDTA (50 *μ*L) was used for peripheral hemogram analysis by Beckman Coulter Ac and T 5 full-automatic blood cell analyzer (Beckman, USA). After the mice were sacrificed, the thymus gland and spleen were excised and weighted to calculate the thymus index and spleen index: (organ weight/body weight) × 1000.

### 2.5. Bone Morrow Histological Examination

Three femoral bones in each group were removed from the sacrificed mice and fixed in 4% paraformaldehyde. And then, bones were treated with a formic acid-sodium citrate decalcification solution for 5 days. Bones were embedded in paraffin, sectioned at 5 *μ*m for staining with H&E, to visualize the histological examination of bone marrow.

### 2.6. Cytokines Levels of G-CSF, GM-CSF, IL-3, IL-6, and TNF-*α* in Plasma or Serum

Plasma or serum samples were collected from the sacrificed mice, and the levels of G-CSF, GM-CSF, IL-3, IL-6, and TNF-*α* in plasma or serum were measured by Enzyme-Linked Immunosorbent Assay (ELISA) kits (Beijing Sino-UK Institute of Biological Technology, Beijing, China).

### 2.7. Analysis of G-CSF, GM-CSF, IL-3, IL-6, and TNF-*α* mRNA Expressions in Spleen

The spleen tissues were homogenized and the total RNA was extracted from the supernatant fraction. Then total RNA from each sample was reverse-transcribed into cDNA using a Super RT cDNA kit (Thermo, USA), and the synthesized cDNA was used for RT-qPCR amplification using SYBR green Real-time PCR Master Mix. Furthermore, the nucleotide sequences of forward and reverse primers used for PCR are shown in Table 1. The cycling conditions were 95°C for 10 min, followed by 40 cycles of 95°C for 15 s, 60°C for 60 s, and 75°C for 20 s. The RT-qPCR analysis was performed with the Light Cycler 480 RT-qPCR System. The results of relative expression of mRNA in each group were semiquantitated using the comparative *C*_*t*_ method and calculated setting normal control as 1.

### 2.8. Western Blot Analysis (G-CSF, GM-CSF, IL-3, IL-6, and TNF-*α*) in Bone Marrow

Three mice in each group were sacrificed and then bone marrow from the femur was collected. The total protein in bone marrow was extracted with lysis buffer (50 mM Tris, pH 8.0, 150 mM NaCl, 0.1% sodium dodecyl sulfate, 0.5% sodium deoxycholate, 100 mg/mL phenylsulfonyl fluoride, 2 mg/mL aprotinin, 1 mg/mL pepstatin, and 10 mg/mL leupeptin), and 50 mg protein was resolved on a 10% sodium dodecyl sulfate polyacrylamide gel. The fractionated proteins were electrophoretically transferred to an immobilon polyvinylidene difluoride membrane and probed with antibody of G-CSF, GM-CSF, IL-3, IL-6, and TNF-*α* (Bioss. Inc., Beijing, China).

### 2.9. Statistical Analyses

Results are expressed as mean ± SD. Statistical significant differences were determined by one-way analyses of variance and Student's *t*-tests. A *P* value < 0.05 indicates a statistically significant difference.

## 3. Results

### 3.1. Effects of PF and AF on Peripheral Blood Cells

As shown in Table 2, the number of white blood cells (WBC) in irradiation-induced model group was significantly reduced compared to that in normal group (*P* < 0.001). PF-H or AF-H treatment significantly elevated the number of WBC (*P* < 0.001). AF-L group also increased the number of WBC significantly (*P* < 0.01).

### 3.2. Effects of PF and AF on the Change of Body Weight, Thymus Index, and Spleen Index

**Figure 2:**
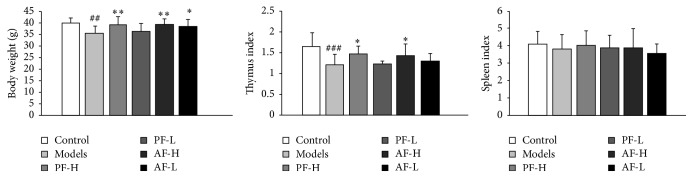
Effects of PF and AF on body weight, thymus index, and spleen index. Control = negative control (with same volume of physiologic saline). Data are expressed as means ± SD (*n* = 10). Compared with the control group: ^##^ *P* < 0.01 and ^###^ *P* < 0.001; compared with the model group: ^*∗*^ *P* < 0.05 and ^*∗∗*^ *P* < 0.01.

As shown in Figure 2, the body weight and thymus index in model mice were significantly decreased by the irradiation treatment (*P* < 0.01, *P* < 0.001). PF-H significantly increased the body weight (*P* < 0.01) and thymus index (*P* < 0.05) and AF-H also significantly increased the body weight (*P* < 0.01) and thymus index (*P* < 0.05). AF-L group increased the body weight (*P* < 0.05). It proved that both PF and AF could reverse the loss of body weight and the atrophy of hemopoietic organ (also known as immune organs) induced by irradiation. There is no change on spleen index.

### 3.3. Effects of PF and AF on Bone Marrow Histopathology

**Figure 3:**
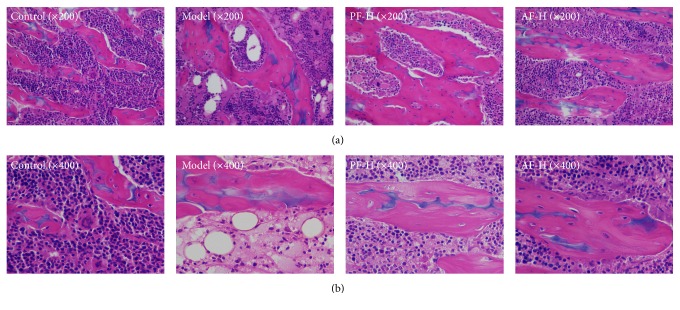
Effects of PF and AF on the bone marrow tissue histomorphology of femoral bone (H&E staining. (a): 1 × 200, (b): 1 × 400).

As shown in Figure 3, the color of the bone marrow tissue of normal mice was uniform, and the architectures of periosteum, cavitas medullaris, and cartilage cells were clear, whereas, the bone marrow of model group showed a great deal of nucleated myelocytes that was reduced and replaced by vacuolation compared with that of the normal group. PF-H groups decreased the vacuole-like degradation clearly. AF-H also could increase the cell density and decrease the number of vacuole-like degradation.

### 3.4. Effects of PF and AF on the Hematopoiesis-Related Cytokines

As shown in Table 3, compared to the control group, serum level of GM-CSF and plasma level of G-CSF were significantly decreased after irradiation (*P* < 0.001, *P* < 0.001), while levels of GM-CSF and G-CSF in PF-H (*P* < 0.001, *P* < 0.001) and AF-H significantly increased (*P* < 0.001, *P* < 0.001), respectively. However, PF-L significantly increased G-CSF (*P* < 0.01) and AF-L increased GM-CSF (*P* < 0.01). Irradiation induced a decrease of IL-3 in serum (*P* < 0.001), and PF-H and AF-H administration increased the level of IL-3 compared to the model (*P* < 0.001, *P* < 0.001). As shown, irradiation also induced an increase of TNF-*α* compared to control (*P* < 0.01), and PF-H and AF-H administration decreased the level of TNF-*α*, respectively (*P* < 0.01, *P* < 0.01).

### 3.5. Effects of PF and AF on G-CSF, GM-CSF, IL-3, IL-6, and TNF-*α* mRNA Levels

**Figure 4:**
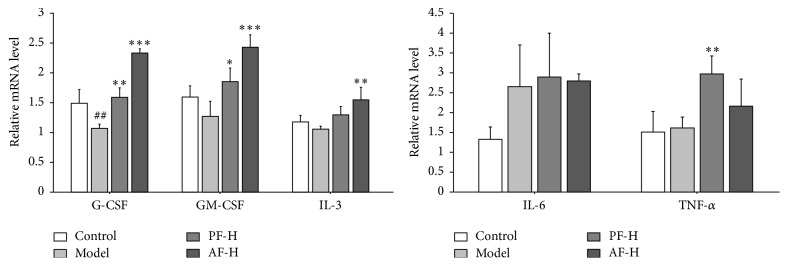
Effects of AF on GM-CSF, G-CSF, IL-3, IL-6, and TNF-*α* mRNA levels. Control = negative control (with same volume of physiologic saline). Data are expressed as means ± SD (*n* = 3). Compared with the control group: ^##^ *P* < 0.01; compared with the model group: ^*∗*^ *P* < 0.05, ^*∗∗*^ *P* < 0.01, and ^*∗∗∗*^ *P* < 0.001.

As shown in Figure 4, compared with the control group, the mRNA level of G-CSF was significantly decreased (*P* < 0.01) in the model animals. Compared to the model group, the mRNA level of G-CSF was elevated in PF-H (*P* < 0.01) and in AF-H (*P* < 0.001) treatment groups. Besides, there were no significant differences between the normal and the model mice on the mRNA levels of GM-CSF, IL-3, IL-6, and TNF-*α*. PF-H increased the mRNA levels of GM-CSF and TNF-*α* (*P* < 0.05, *P* < 0.01). AF-H significantly increased the mRNA levels of GM-CSF and IL-3 (*P* < 0.001, *P* < 0.01). The data suggested that PF-H and AF-H could significantly enhance the mRNA levels of GM-CSF, G-CSF, and TNF-*α* in the spleens.

### 3.6. Effects of PF and AF on GM-CSF, G-CSF, IL-3, IL-6, and TNF-*α* Protein Levels

**Figure 5:**
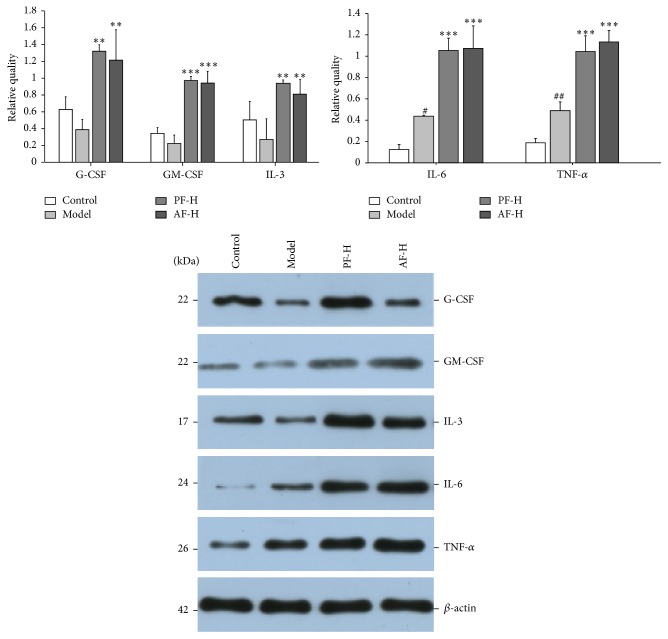
Effects of PF and AF on GM-CSF, G-CSF, IL-3, IL-6, and TNF-*α* protein levels. Control = negative control (with same volume of physiologic saline). Data are expressed as means ± SD (*n* = 3). Compared with the control group: ^#^ *P* < 0.05 and ^##^ *P* < 0.01; compared with the model group: ^*∗∗*^ *P* < 0.01 and ^*∗∗∗*^ *P* < 0.001.

As shown in Figure 5, irradiation induced a decrease of G-CSF, GM-CSF, and IL-3 at protein levels compared to the control group, although there were no significant differences. PF-H could increase the levels of G-CSF, GM-CSF, and IL-3 compared to the control group, respectively (*P* < 0.01, *P* < 0.001, and *P* < 0.01) and AF-H also could increase the levels of G-CSF, GM-CSF, and IL-3 compared to the control group, respectively (*P* < 0.01, *P* < 0.001, and *P* < 0.01). As shown, irradiation induced increases of IL-6 and TNF-*α* after irradiation compared to the sham irradiation (*P* < 0.05, *P* < 0.01), PF-H could increase the levels of IL-6 and TNF-*α* compared to model group (*P* < 0.001, *P* < 0.001), and AF-H significantly increased IL-6 and TNF-*α* levels compared to control group (*P* < 0.001, *P* < 0.001). Interestingly, the IL-6 and TNF-*α* protein levels were opposite to those in serum.

## 4. Discussion

Mice exposure to ionizing radiation leads to injury to the lymphoid and haemopoietic system, which can result in septicaemia and death [16]. Myelosuppression is a common side effect of radiotherapy and bone marrow hemopoietic tissue is the most sensitive to radiation [19]. Previous studies suggested irradiation inducing myelosuppression syndrome, capable of agent enhancing survival typically being associated with accelerated hematopoietic cytokines regeneration. Peripheral blood cell is derived from the proliferation and differentiation of hemopoietic stem cells and progenitor cells and bone marrow is in charge of the peripheral blood cells continuous replenishment [20]. In this study, the number of peripheral blood cells indirectly reflects the overall hematopoietic function. Firstly, results (Table 2) showed that PF and AF could significantly reverse the decrease of the quantities of WBC; the effects of PF and AF on peripheral blood cells are consistent with the previous published results of CPA [14]. Secondly, the pathological sections show that AF had improvement effects on the histopathology of bone marrow tissue (Figure 2). Thirdly, body weight was decreased in model group, while the decrease was reversed in the AF-treated mice, which means that PF and AF play a positive role in preventing body weight loss resulting from irradiation. Finally, it is well known that thymus and spleen are important hematopoietic organs besides bone marrow [21]. The results (Table 3) showed that PF and AF increased the thymus indices, which implied that PF and AF possess the capacity of alleviating the loss of body weight and the atrophy of hemopoietic organs (also known as immune organs) induced by irradiation besides promoting bone marrow hematopoietic function. It was exactly consistent with the previous published results of CPA or Fu fang E'jiao Jiang (a famous TCM formula, which has widely been used in treating anemia) [14, 16]. The results demonstrate that PF and AF also have protection effects on the immune conditions against the side effects caused by radiotherapy.

In addition, hematopoiesis is a diverse process, regulated by various hematopoietic cytokines, such as G-CSF, GM-CSF, IL-3, IL-6, and TNF-*α*. A key feature of enhancing recovery from the radiation syndrome is the hematopoietic cytokines activation [22]. In this study, both PF and AF improved the G-CSF level in plasma, mRNA level in spleen, and protein level in bone marrow cells. The effect on G-CSF was consistent with the previous published results demonstrating the effect of Siwu-Tang in anaemia model by irradiation [16]. We also analyzed the contents of PF and AF in Siwu-Tang by high-performance liquid chromatography (HPLC) analysis, which showed the presence of PF and AF, which showed the presence of PF and AF to be 6.4% and 3.3%, respectively. Although the activities of a single compound might not show the same activities of a formula, our results suggest that the majority of effective ingredients in Siwu-Tang are PA and AF. Moreover, both PF and AF increased the levels of GM-CSF and IL-3 in serum and increased the protein levels in bone barrow cells, and AF enhanced the IL-3 mRNA levels in spleen. The reason may be that PF and AF regulated the GM-CSF and IL-3 to stimulate proliferation and maturation of granulocyte and macrophage myeloid cells [23] and accelerate the recovery of circulating hematopoietic lineage and stimulate the growth and effector functions of lymphocytes and macrophages [24]. Furthermore, TNF-*α* is a potent inhibitor, which in turn affects the differentiation of early bone marrow progenitor cells by altering their response to CSFs [25] and accelerating the release of granulocytes from bone marrow [26]. Interestingly, our present study revealed that both PF and AF could enhance the mRNA level and protein level, while the protein levels of TNF-*α* were opposite to those in serum, and the reason needs to be investigated further. However, IL-6 is produced by a number of normal and transformed cell lines [27], which can either promote or inhibit the growth of tumor cells. Importantly, IL-6 acts in concert with IL-3 to induce multilineage progenitors from murine spleens [28]. However in our study, no significant activation of IL-6 was observed. Similar results showed that Rg1 (a chemical compound in* Panax ginseng*, which is traditionally used as a restorative, antidiabetic, antivomiting, and anticancer agent worldwide) or Fu fang E'jiao Jiang (*Panax ginseng* is a component herb of this formula) changed the expression of hematopoiesis-related cytokines in animal models [19, 27]. PF and AF promoted hematopoiesis probably through stimulating the expression of GM-CSF, IL-3, and G-CSF in myelosuppression mice induced by irradiation. Further experiments are necessary to clarify their mechanism of action. The effect of PF and AF on other hematopoiesis-related cytokines remains to be explored further in the ongoing study.

## 5. Conclusions

In conclusion, the results of the present study indicate that PF and AF have potent ameliorative effects on radiation-induced myelosuppression mice. PF and AF stimulate the hematopoiesis-related cytokines and enhanced hematopoietic progenitor cell activity resulting in accelerated blood cell recovery. Additionally, further pharmacological studies to determine more mechanisms underlying the therapeutic effects of PF and AF are needed.

## Figures and Tables

**Table 1:** Primers used for quantitative RT-PCR.

Genes	Forward (5′–3′)	Reverse (5′–3′)
GAPDH	AGGAGCGAGACCCCACTAACA	AGGGGGGCTAAGCAGTTGGT
IL-3	GCCTGCCTACATCTGCGAAT	GGTTAGGAGAGACGGAGCCA
IL-6	GTCCGGAGAGGAGACTTCAC	CTGCAAGTGCATCATCGTTGT
GM-CSF	TTACTTTTCCTGGGCATTGTGG	CAGGAGGTTCAGGGCTTCTTTG
TNF-*α*	ACCCTCACACTCACAAACCA	ATAGCAAATCGGCTGACGGT
G-CSF	CGCATGAAGCTAATGGGTGAGT	GACGGGTCTGAGGCACTTGTT

**Table 2:** Effects of PF and AF on peripheral blood cells (means ± SD, *n* = 10).

Groups	WBC	RBC	HGB
Control	7.50 ± 1.35	2.67 ± 0.65	102.3 ± 8.99
Model	1.23 ± 0.52^###^	2.23 ± 0.47	79.2 ± 11.33^###^
PF-H	2.40 ± 0.47^*∗∗∗*^	2.36 ± 0.96	87.0 ± 5.29
PF-L	1.47 ± 0.26	2.24 ± 0.53	83.1 ± 11.40
AF-H	2.37 ± 0.41^*∗∗∗*^	2.39 ± 0.62	87.5 ± 6.87
AF-L	2.07 ± 0.46^*∗∗*^	2.14 ± 0.41	84.9 ± 10.10

Note: compared with the control group: ^###^ *P* < 0.001; compared with the model group: ^*∗∗*^ *P* < 0.01 and ^*∗∗∗*^ *P* < 0.001.

**Table 3:** Effects of PF and AF on the hematopoiesis-related cytokines levels (means ± SD, *n* = 10).

Groups	GM-CSF	G-CSF	IL-3	IL-6	TNF-*α*
Control	115.58 ± 16.79	122.74 ± 12.86	18.26 ± 1.43	104.08 ± 16.94	56.72 ± 7.31
Model	74.83 ± 13.60^###^	85.83 ± 15.79^###^	11.01 ± 1.53^###^	154.90 ± 24.37^###^	70.02 ± 11.03^##^
PF-H	105.06 ± 18.12^*∗∗∗*^	119.31 ± 15.98^*∗∗∗*^	14.69 ± 2.56^*∗∗∗*^	130.62 ± 33.55	54.15 ± 7.47^*∗∗*^
PF-L	85.41 ± 12.36	107.01 ± 14.54^*∗∗*^	12.43 ± 1.53	131.35 ± 32.07	62.24 ± 14.04
AF-H	112.94 ± 19.31^*∗∗∗*^	117.12 ± 21.86^*∗∗∗*^	14.80 ± 2.69^*∗∗∗*^	129.22 ± 34.48	53.55 ± 10.95^*∗∗*^
AF-L	94.27 ± 14.61^*∗∗*^	88.90 ± 12.72	12.16 ± 1.69	152.08 ± 26.75	67.11 ± 10.94

Note: compared with the control group: ^##^ *P* < 0.01 and ^###^ *P* < 0.001; compared with the model group: ^*∗∗*^ *P* < 0.01 and ^*∗∗∗*^ *P* < 0.001.
